# Direct *in vitro* propagation of avian germ cells from an embryonic gonad biorepository

**DOI:** 10.1016/j.psj.2024.104260

**Published:** 2024-08-26

**Authors:** Tuanjun Hu, Phillip H. Purdy, Marcel H. Blank, Christine K. Muhonja, Ricardo J.G. Pereira, Christian K. Tiambo, Mike J. McGrew

**Affiliations:** ⁎ The Roslin Institute, University of Edinburgh, Easter Bush Campus, Edinburgh, EH25 9RG, UK; † National Gene Pool of Waterfowl, Jiangsu Agri-Animal Husbandry Vocational College, Taizhou China 225300; ‡ United States Department of Agriculture (USDA), Agriculture Research Service (ARS), National Animal Germplasm Program, Fort Collins, CO, 80521-4500, USA; § Department of Animal Reproduction, College of Veterinary Medicine and Animal Science, University of São Paulo, Pirassununga, Sao Paulo, 13635-900, Brazil; ║ Centre for Tropical Livestock Genetics and Health (CTLGH), International Livestock Research Institute (ILRI), Box 30709, Nairobi, Kenya; ¶ Kenya Agricultural and Livestock Research Organization (KALRO), P. O. Box 25, Naivasha, Kenya

**Keywords:** primordial germ cell, gonad, avian, chicken, cryopreservation

## Abstract

Direct introduction of cryopreserved embryonic gonadal germ cells (**GGC**) into a sterile chicken surrogate host to reconstitute a chicken breed has been demonstrated as a feasible approach for preserving and utilizing chicken genetic resources. This method is highly efficient using male gonads; however, a large number of frozen female embryonic gonads is needed to provide sufficient purified GGC for the generation of fertile surrogate female hosts. Applying this method to indigenous chicken breeds and other bird species is difficult due to small flock numbers and poor egg production in each egg laying cycle. Propagating germ cells from the frozen gonadal tissues may be a solution for the biobanking of these birds. Here, we describe a simplified method for culture of GGC from frozen embryonic 9.5 d gonads. At this developmental stage, the germ cells are autonomously shed into medium, yielding hundreds to thousands of mitosis-competent germ cells. The resulting cultures of GGC have over 90% purity, uniformly express SSEA-1 and DAZL antigens and can re-colonize recipient's gonads. The GGC recovery rate from frozen gonads are 42% to 100%, depending on length of cryopreservation and the breed or line of chickens. Entire chicken embryos can also be directly cryopreserved for later gonadal isolation and culture. This storage method is a supplementary approach to safeguard local indigenous chicken breeds bearing valuable genetic traits and should be applicable to the biobanking of many bird species.

## INTRODUCTION

Primordial germ cells (**PGC**) are embryonic undifferentiated progenitor cells that develop into functional gametes and transmit the parental genetic information to the next generation. The specification of avian PGCs relies upon cell autonomous preformation using a maternally deposited germ plasm (Tsunekawa et al., 2000; Naito et al., 2001). In contrast to other vertebrates, the avian PGC enter the blood vessels and migrate along the blood circulation in early-stage embryos until reaching the lateral plate. From there, they colonise the gonadal anlagen and proliferate during embryonic stages to form the resident germ cell population (Nieuwkoop and Sutasurya, 1979). This characteristic offers a great opportunity for the manipulation of the avian germ cell lineage by either retrieving embryonic blood for *in vitro* PGC culture or by introducing exogenous PGCs into the vascular system of a host for generation of chimeric birds (reviewed in Woodcock et al., 2017).

The *in vitro* culture of chicken PGC has advanced, especially following the success in developing a defined serum-free, low calcium culture medium (Whyte et al., 2015) which simplifies culturing in comparison to the original medium containing sera and co-culture with feeder cells (van de Lavoir et al., 2006). Progress on the sterilization of surrogate host embryos resulted in reduced endogenous PGC competition, increased efficacy of the desired exogenous germ cell transmission, and facilitated the application of PGC-based techniques. To accomplish this, chemical (busulfan, Lee et al., 2013) and radiation methods (Nakamura et al., 2012; Macdonald et al., 2010) were developed to ablate endogenous PGCs. Alternatively, to eliminate the harmful effects of these treatments on surrogate hosts, such as low hatchability of chimeric chicks, recently, 2 types of genetically sterile surrogate hosts were made by ‘knock out’ of the germ cell developmental gene, DDX4 (Taylor et al., 2017; Woodcock et al., 2019) or the recombination, ‘knock in’, of a chemical inducible apoptotic Caspase 9 reporter gene into the germ cell specific DAZL locus (Ballantyne et al., 2021). The endogenous PGCs in these hosts can be completely depleted from both hosts and only exogenous PGCs allowed to transmit to their progeny. Additionally, the improvement of a microinjection technique for introducing PGCs into surrogate embryos, such as *in ovo* injection through a small window on the eggshell instead of *ex ovo* injection and culture in surrogate eggshells during embryo development, improves the generation of healthy live birds hatched from manipulated embryos (Idoko-Akoh and McGrew, 2023). The advancement of these techniques aided developments in avian transgenesis and cryopreservation of chicken germplasm.

Poultry species are kept as live flocks for maintenance of breeds because biobanking of avian germplasm is problematic. Climate change and urbanization are shrinking the habitats of local chicken breeds and other avian species, and epidemic diseases, such as avian influenza endanger avian species. Also, specialized research breeds of chicken housed at research institutes are being lost due to the costs of maintaining these lines *in situ* (Fulton and Delany, 2003). Sperm cryopreservation is an effective method for preservation of the genetic diversity of avian species, and artificial insemination is a noninvasive method for fertilizing hens. However, success with artificial insemination when frozen-thawed rooster sperm is used is breed dependent. Moreover, the full genetic background is not recovered using frozen-thawed sperm because roosters are homogametic (ZZ), the W sex chromosome is only carried by the female, and the hatchability rates of eggs from hens artificially inseminated with frozen-thawed rooster sperm is often low to very low (WZ, Hart-Johnson and Mankelow, 2022, Whyte et al, 2016).

Cultured PGCs isolated from the embryonic blood or germinal crescent offers a more effective method for biobanking of chicken breeds, due to indefinite expansion of PGC numbers *in vitro* (Nandi et al., 2016). However, obtaining embryos at the correct developmental stage using eggs from local breeds, which are kept in small flocks and yield low numbers of eggs, is prohibitive and this technique requires technical resources for isolation and culturing of the cells. Cryopreserving reproductive tissues (ovaries and testes) from newly hatched chicks have been demonstrated as feasible approach for germline transmission from frozen donor tissues via chimeric recipient hosts. However, these approaches are technically demanding because a special surgical procedure is required to transplant the frozen tissues into a recipient, due to avian reproductive organs laying inside the abdominal cavity, and immunosuppressive drugs are often used to reduce rejection of the transplanted tissues (Blackburn et al., 2023).

Cryopreserving embryonic gonads is an alternative method (Tajima et al., 1998). Germline transmission has been demonstrated from germ cells isolated from embryonic gonads (reviewed by Nakamura et al., 2013). Furthermore, we demonstrated that direct introduction of frozen-thawed gonadal germ cells (**GGC**) isolated from embryos at a later developmental stage (embryo d 9-10) into sterile hosts could directly produce large numbers of pure offspring containing multiple genotypes (Hu et al., 2022). However, many female donor gonads are needed to achieve robust laying from surrogate host hens.

Here, we demonstrate a simplified method to culture chicken germ cells from cryopreserved embryonic gonads. This method will be suitable for the cryopreservation of local, indigenous breeds of chicken in locations that lack resources for the immediate *in vitro* culture of PGCs

## MATERIALS AND METHODS

### Chicken Embryos and Germ Cell Culture Medium

Fertile eggs were obtained from the National Avian Research Facility (**NARF**) at The Roslin Institute UK (www.ed.ac.uk/roslin/national-avian-research-facility). The chicken breeds used included 2 transgenic lines on a Hyline Brown layer background: GFP-transgenic line (ubiquitious expressing GFP protein) (Macdonald et al., 2012), iCaspase 9-GFP transgenic chicken line (germ cells specifically labelled with a DAZL-GFP reporter) (Ballantyne et al., 2021). The SPF White leghorn inbred line N (bred on a single MHC haplotype for pathogen resistance), the Light Sussex breed, the line J chicken (originally selectively bred from Brown Leghorn chickens to study a variety of traits, e.g. egg laying, plumage, and vigour), Hyline commercial layer line, and a local Kenyan ecotype from Laikipia. The eggs were incubated in a rocking incubator at 37.8°C with 60% humidity for 8 – 10 d. All animal management, maintenance, and embryo manipulations were carried out under UK Home Office license and regulations. Experimental protocols and studies were approved by the Roslin Institute Animal Welfare and Ethical Review Board Committee and by the International Livestock Research Institute (ILRI) Animal Care and Use Committee (IACUC).

Avian germ cell culture medium (FAOT medium) contained 1  ×  B-27 supplement (Thermo Fisher Scientific), 2.0 mM GlutaMax (Thermo Fisher Scientific), 1  ×  nonessential amino acids (Thermo Fisher Scientific), 1  ×  EmbryoMax nucleosides (Merck Millipore), 0.1 mM β-mercaptoethanol (Thermo Fisher Scientific), 0.2% ovalbumin (Sigma), 1.2 mM pyruvate (Thermo Fisher Scientific), 0.15 mM CaCl2, 0.01% sodium heparin (Sigma) in DMEM, a custom basal medium (a modification of knockout DMEM [250 mosmol/L, 12.0 mM glucose, and CaCl_2_-free; ThermoFisher Scientific]). The following growth factors were added before use: human Activin A, 25 ng/mL (Peprotech); human FGF2, 4 ng/mL (R&D Biosystems); 0.2% chicken serum (Biosera).

### Culturing Germ Cells From Frozen Gonadal Tissues

The process for dissection and cryopreservation of gonads from d 9-10 embryos were performed as described previously (Hu et al., 2022). Male and female gonads are clearly different at this development stage and can be sexed visually; male embryo gonads are almost equal in size and sausage shaped, female embryo gonads are asymmetrical with the female left gonad being much larger with a flattened shape.

Freezing whole (d 8-10 of incubation) embryos for cryopreservation of gonads was also tested. Briefly, after cracking open the eggshell and removing shell membranes, embryos were decapitated and placed in a 100 mm Petri dish. Neck tissue, wings and legs were removed and embryos were washed 3 time with PBS. The trimmed embryo body was transferred into a 1.8 mL cryovial, prefilled with 650 uL of STEM-CELLBANKER, 3 embryos per tube. Before transferring embryo bodies into cryotube, several small holes were made gently in each embryo with a 23G hypodermic needle to help the perfusion of cryopreservation medium into embryo tissue. After 50 min equilibration at room temperature, the cryovials were placed into a Mr. Frosty Freezing Container and put into -80°C freezer overnight. The vials were moved into a -150°C freezer the next day. Samples were stored between 7-30 d.

Frozen gonads were thawed at 37°C for 10 s until ice has disappeared, the tissues transferred from a cryotube into a 1.5 mL screw-cap Eppendorf tube and briefly centrifuged at 2,000 rpm for 4 s. The cryo medium (STEM-CELLBANKER) was removed and replaced with 1 mL of FAOT medium. The tube was set on ice for 30 min to equilibrate the tissues and the medium with tissues were then transferred into a sterile petri dish using P1000 Eppendorf pipette. Under a stereo microscope in a biosafety hood, one gonad was picked up using a sterile hypodermic needle and transferred into a well on a 48-well plate prefilling with 300 µL FAOT medium with 0.2% chicken serum. For frozen whole embryo body, the vials were thawed at 37°C for 150 s in a water bath, the embryo body was place into a 100 mm Petri dish for dissection of gonadal tissue as described before (Hu et al., 2022). The individual gonad was then directly transferred into FAOT medium with 0.2% chicken serum on a 48-well plate. The plate was returned into a tissue culture incubator at 37°C with 5% CO_2_.

The following day (about 20 h in culture), the gonadal tissue was gently washed several times using the growth medium in the well and a P1000 Eppendorf pipette to remove germ cells adherent on the tissue pieces. The cell suspension was then carefully aspirated (but not tissue clumps) and transferred into a new well on a 48 well plate. One-third of the medium in the new well was changed every 2 d until the cell number reached over 1 × 10^5^ cells/mL (cut-off cell number for a successful derivation).

### Characterization of Cultured GGC

The cell growth rates were analyzed by seeding 3 × 10^4^ cells into 500 mL FAOT medium in 1 well on a 24-well plate. A total of 8 GGC derivations from either line N or Light Sussex female embryos were analyzed. The cell numbers were first counted on d 4. After counting, all cells were re-seeded into the same well with fresh medium and the cell number was counted again on d 6 and 8 using the same procedure.

The homogeneity of cultured GGC was analyzed by flow cytometry. For GGC derivations from iCaspase 9 embryos, the GFP^+^ PGCs cells were quantitated for GFP fluorescence using flow cytometry, GGC derivations from wild type embryos were used as a negative control. For GGC derviations from line N or Light Sussex, the cells were immunostained by SSEA-1 (1:500 dilution, Abcam) for 20 min on ice, followed by a AF569-conjugated anti-mouse IgM antibody (1:5000 dilution, Life technology). The resulting cells were resuspended in 300 μL PBS with SYTOX® Blue Dead Cell Stain (1.0 μM, Invitrogen) 5 min prior to analysis using a BD LRSFortessa flow cytometer (BD Biosciences, UK). At least 100,000 events were acquired. Dead cells were excluded by SYTOX Blue staining and doublets were discriminated based on signal processing (SSC-A/H or FSC-A/H). Data were analysed using FlowJo software (FlowJo, Ashlan, OR).

Fluorescence imaging was performed on GGC derivations from line N or Light Sussex embryos. The cells were fixed in 4% PFA for 20 min and then permeabilized by 0.1% Triton X-100 for 15 min. After blocking in 2% goat serum for 30 min, the cells were incubated with anti-DAZL (1:250 dilution, Abcam) and anti-SSEA-1 (1:250 dilution, R&D system) antibody cocktail for 1 h, followed by AF488-conjugated anti-rabbit (1:500 dilution, Life technology) and AF546-conjugated anti-mouse IgM (1:1000 dilution, Life technology) antibody cocktail for 30 min, to identify cells co-labelled with anti-DAZL and anti-SSEA-1.

The capability of GGCs to re-colonise host gonads was tested by injection of male or female GGC derivations expressing GFP protein into non-GFP inbred line N embryos at 2.5 d (HH16) through a small window opened at the blunt end of the egg. Each egg was injected with 1 × 10^4^ cells in 1 µL B-27 DMEM basal medium into the dorsal aorta. After injection, the window was sealed with clear adhesive packaging tape and the eggs were incubated without rocking for 7 d. Donor cell colonization of recipient embryonic gonads was visualized using a eGFP filter on a Zeiss Axiozoom V16 microscope.

### Statistics

The statistical analyses were calculated using a 2-tailed Student's *t*-test. The error bars in all figures are S.E.M. It is considered statistically significant when *P* < 0.05.

## RESULTS

### Isolation and Culture of Germ Cells From Frozen Embryonic Gonads

**Figure 1:**
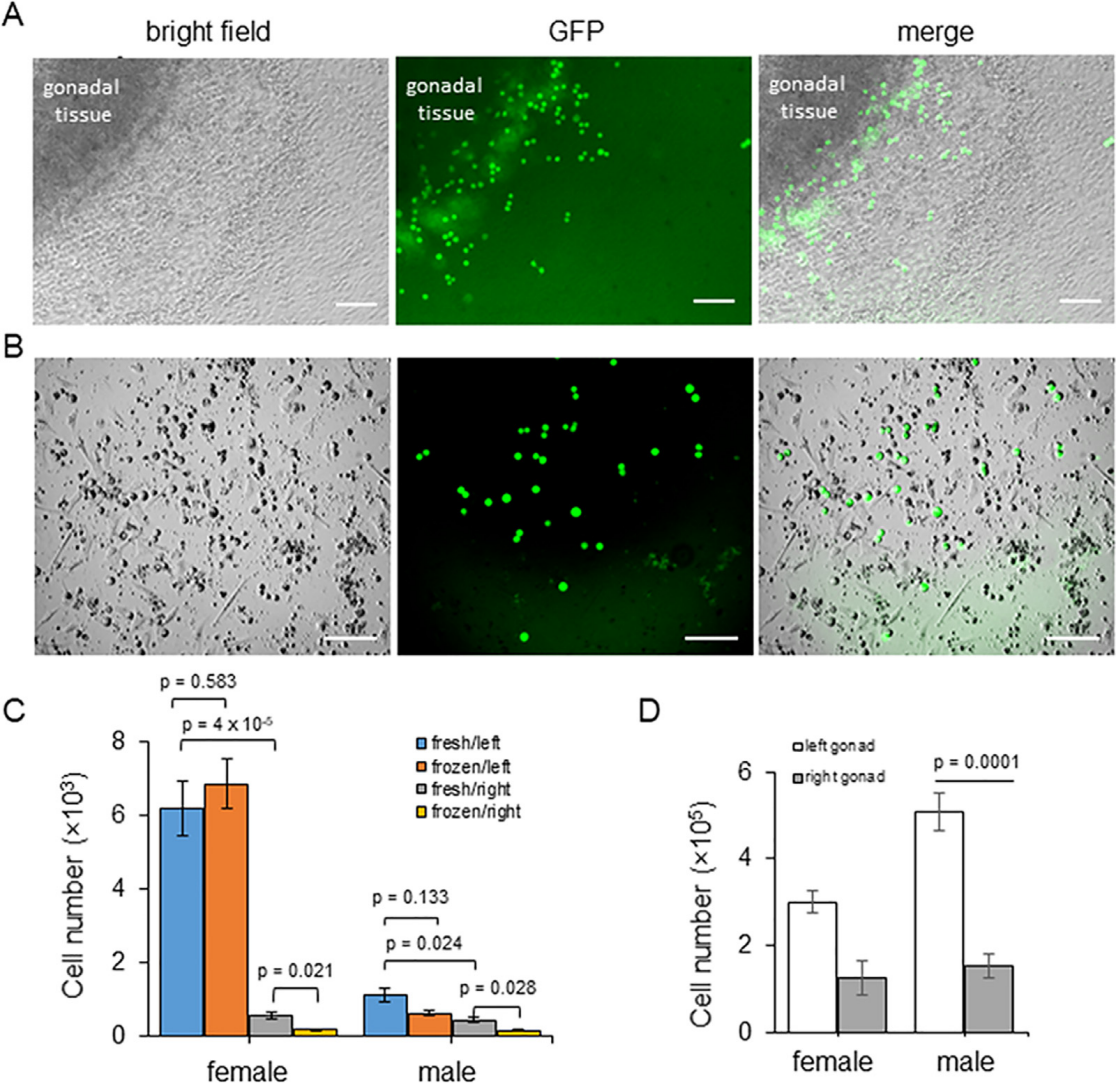
Isolation and culture of germ cells from frozen gonads, from d 10 iCaspase 9-GFP embryos. (A) Gonads were cultured for 2 d. GFP^+^ germ cells are observed migrating from the gonadal tissue. Scale bar = 100 µm. (B) After 20 h culture, the GFP^+^ germ cells migrate from the tissue and become fully detached suspension cells. GFP^-^ cells are observed attached to the substrate. Scale bar = 100 µm. (C) Number of gonadal germ cells shed into culture medium. After 20 h of culture, GFP^+^ cells in medium from cultured left or right gonads were counted. The data represent average number of germ cells yielded from single gonads. About 5 to 6 pools of gonads freshly isolated from iCaspase 9 embryos at 10 d incubation, 5 to 11 gonads in each pool were tested, n = 2 repeats; the frozen tissues from same age of iCaspase 9 embryos were stored at −150°C for >10 months, 3 to 4 pools of 5 to 7 gonads from each pool were tested, experiment performed once, the error bar is S.E.M., *P*-value by Student's *t*-test. (D) Culture of germ cells from frozen gonads stored (−150°C) for over 7 months. The left or right gonadal tissue from each embryo was separately cultured for 15 d. (female right gonads for 20 d), the cells were counted until number reaching over 1 × 10^5^ cells for each derivation. The data represent average from 6 pairs of male gonads and 9 pairs of female gonads, error bar is S.E.M., *P*-value by Student's *t*-test.

Chicken gonadal germ cells have been shown to be spontaneously released from embryonic d 7 gonads when placed in low calcium saline buffer (PBS) (Nakajima et al., 2011; Nakajima et al., 2022). Therefore, we tested if germ cells would be released from both fresh and cryopreserved gonads into low calcium PGC culture medium (Whyte et al., 2015) using a transgenic chicken line (iCaspase9-GFP), containing an iCaspase 9 and GFP reporter construct targeted to the DAZL locus. This transgenic line specifically expresses GFP in germ cells at all developmental stages which provides a useful tool to study the migratory characteristics of embryonic germ cells (Ballantyne et al., 2021). Pairs of female gonads were dissected from d 9 iCaspase9-GFP embryos and the whole tissues were transferred into FAOT culture medium. During a 48 h period, GFP^+^ GGCs can be clearly visualized migrating from the gonads. In culture, these putative germ cells loosely adhered to the gonadal somatic cells at the bottom of the well (Figure 1A) before transitioning to suspension cells (Figure 1B). We counted the number of GFP^+^ germ cells present after 20 h culture, to estimate the seeding potential of individual gonads. As shown in Figure 1C, each left female gonad yielded over 6,000 GFP^+^ cells. This number was 10-fold higher than cells yielded from the right female gonad (580 cells, *P* = 4 × 10^-5^). In contrast, male gonads produced fewer suspension GFP^+^ cells. Each left male gonad yielded approximately 1,100 germ cells, which was 3 times higher than the number of germ cells obtained from each right male gonad (400 cells, *P* = 0.024). We next tested the number of GFP^+^ cells obtained from cryopreserved gonads. We observed GFP^+^ germ cells were autonomously shed into the growth medium when frozen (>10 months in −150°C) gonadal tissues were placed in culture medium for 20 h. Similarly, the frozen left gonads from both sexes produce more germ cells than the right gonads and yielded statistically comparable numbers of cells as that of fresh tissues. However, the freeze/thaw process decreased the germ cell yield from the frozen right gonadal tissues when compared with fresh tissues (Figure 1C). We next cultured gonadal germ cells from frozen tissues from d 10 iCaspase 9 embryos (>7 months in −150°C) to test if the germ cells discharged from 20 h culture can be cultured in defined medium FAOT until sufficient numbers for biobanking (>10^5^ cells) are obtained. After 15 d in culture (except for cells from female right gonads for 20 d), the number of GGCs from left gonads is much higher than that from right gonads for both sexes. Interestingly, although the cell seeding number is much lower for male left gonads, they produced more than the female left gonads in total cells yielded (Figure 1D). Although the initial cell number could be a main reason for this difference, the physiological differences between the left and right GGCs might also affect the cell growth at the beginning of *in vitro* culture.

We next tested these culture conditions on 4 research chicken lines and 2 local breeds, and on gonadal tissues cryopreserved (−150°C or liquid nitrogen) for different periods of time (Table 1). As expected, using freshly isolated gonadal tissues a high number of GGC cultures were derived from both left and right gonads and after 2 to 3 wk culture produced over 1 × 10^5^ germ cells (per gonadal culture). After 9 months of cryopreservation, the derivation rate is still high using gonads from inbred line N (88%) and Light Sussex (63%) For these cultures, 14 of 16 or 10 of 16 gonads isolated from respectively 8 male line N or Light Sussex embryos successfully generated GGC cultures, with most failed cultures corresponding to the male right gonads. However, it should be noted that cultures from frozen gonads require longer time for expansion to expected cell numbers when comparing with fresh gonads. For tissues in cold storage over 1 year, GGCs were sufficiently cultured from frozen gonadal tissues; however, the derivation rate varied among chicken breeds, high in inbred line N (88%) and transgenic iCaspase 9 (100%), relatively low in transgenic GFP embryos (42%). To test liquid nitrogen storage on GGC culture derivation, gonadal tissues from layer line J stored in liquid nitrogen for 3 years and 2 months were cultured. There was no obvious improvement in GGC derivation rate from these samples, although more studies are needed which include control samples from −150°C storage.

**Table 1:** Gonadal germ cell derivation.

Chicken breed	Embryo sex	Time in cold storage	Germ cell derivation rate (%)	No. of germ cells	No. of successful derivations during each week of culturing					Average derivation culture time ± SD (d)
					≤3 wk	4 wk	5 wk	≥ 6 wk		

iCaspase9	male	0	10/12 (83%)	1.1 × 10^5^	10	–	–	–		18.7 ± 2.21
iCaspase9	male	2 y 4 m	10/10 (100%)	3.5 × 10^5^	10	–	–	–		17.8 ± 5.16
Line N	male	1 y 8 m	14/16 (88%)	3.2 × 10^5^	–	1	4	9		38.8 ± 5.72
Light Sussex	male	9 m	10/16 (63%)	4.1 × 10^5^	–	–	2	8		44.9 ± 6.52
GFP	male	2 y 8 m	5/12 (42%)	4.5 × 10^5^	–	–	2	3		43.2 ± 14.0
Laikipia	male	2 y 6 m	10/10 (100%)	1.1 × 10^6^	–	–	–	10		42 ± 0
Line J*	male	3 y 2 m	5/18 (28%)	3.6 × 10^5^	–	–	–	5		52.2 ± 13.2
iCaspase9	female	0	6/6 (100%, left gonads) 6/6 (100%, right gonads)	1.9 × 10^5^ 2.4 × 10^5^	6 6	– –	– –	– –		16 ± 0 16 ± 0
Line N	female	9 m	17/18 (94%, left gonads) 14/17 (82%, right gonads)	2.6 × 10^5^ 3.1 × 10^5^	– 1	15 –	1 4	1 9		28 ± 8.29 44 ± 11.8
Light Sussex	female	9 m	7/8 (88%, left gonads) 4/8 (50%, right gonads)	3.6 × 10^5^ 1.8 × 10^5^	1 –	– –	2 4	4 –		36.6 ± 8.96 34 ± 2.0
Line J*	female	3 y 2 m	5/10 (50% left gonads) 0/5 (0%, right gonads)	3.0 × 10^5^ –	– –	1 –	– –	4 –		41.2 ± 12.7 –
Laikipia	female	2 y 6 m	8/8 (100% left gonads) 8/8 (100%, right gonads)	1.2 × 10^6^ 9.0 × 10^5^	– –	– –	– –	8 8		42 ± 0 42 ± 0

No.: number.

iCaspase9, GFP: transgenic lines on Hy-line Brown background.

Line N: inbred line on WLH background.

Line J: laying selected line on RIR background.

Light Sussex: UK traditional breed.

Laikipia: local breed from Laikipia County, Kenya.

⁎ Tissues were stored in liquid nitrogen and culture was repeated twice.

We subsequently tested if direct freezing whole embryos is a feasible method for biobanking avian gonadal tissues which may be necessary when sterile dissection conditions are not available. Embryos from d 8, 9 and 10 were cryopreserved, thawed, and gonads were isolated and GGC cultures attempted (Table 2). For Hyline layer eggs, freshly isolated gonads from female embryos at d 8, 9 and 10, achieved 50%, 70% and 60% GGC derivations respectively, and 70%, 100% and 70% respectively for freshly isolated male gonads. In contrast, frozen gonads produce fewer GGC cultures: 10%, 40%, and 0% from female embryos and 30%, 70% and 20% from male embryos at d 8, 9 and 10 respectively. These results indicate that freezing whole d 9 embryos may be an alternative method for cryopreservation of chicken genetic resources.

**Table 2:** Gonadal germ cell derivations from whole cryopreserved embryos.

Embryo age*	Embryo sex	Type of tissue	Germ cell derivation rate (%)	No. of germ cells	No. of successful derivations during each week of culturing				Average derivation culture time (d)
					≤ 3 wk	4 wk	5 wk	≥ 6 wk	
8	Female	fresh†	5/10 (50%)	5.5 × 10^5^	2	2	1	-	26
		frozen	1/10 (10%)	1.8 × 10^5^	-	-	-	1	58
9	Female	fresh	7/10 (70%)	4.4 × 10^5^	4	2	1	-	20
		frozen	4/10 (40%)	1.9 × 10^5^	-	-	-	4	46.5
10	Female	fresh	6/10 (60%)	2.6 × 10^5^	3	3	-	-	22.5
		frozen	0/10 (0)	-	-	-	-	-	-
8	Male	fresh	7/10 (70%)	2.6 × 10^5^	5	2	-	-	20
		frozen	3/10 (30%)	4.8 × 10^5^	-	-	2	1	32
9	Male	fresh	10/10 (100%)	4.6 × 10^5^	6	4	-	-	19
		frozen	7/10 (70%)	2.4 × 10^5^	-		2	5	41
10	Male	fresh	7/10 (70%)	3.3 × 10^5^	4	3	-	-	20
		frozen	2/10 (20%)	2.3 × 10^5^	-	-	-	2	36.5

No.: number.

⁎ Embryos from Hy-line layer line, frozen at −150°C for 7 to 30 d.

† As controls, gonads were isolated from the same stage embryos and directly cultured.

### Propagated GGC Functionally Resemble Propagated PGC

**Figure 2:**
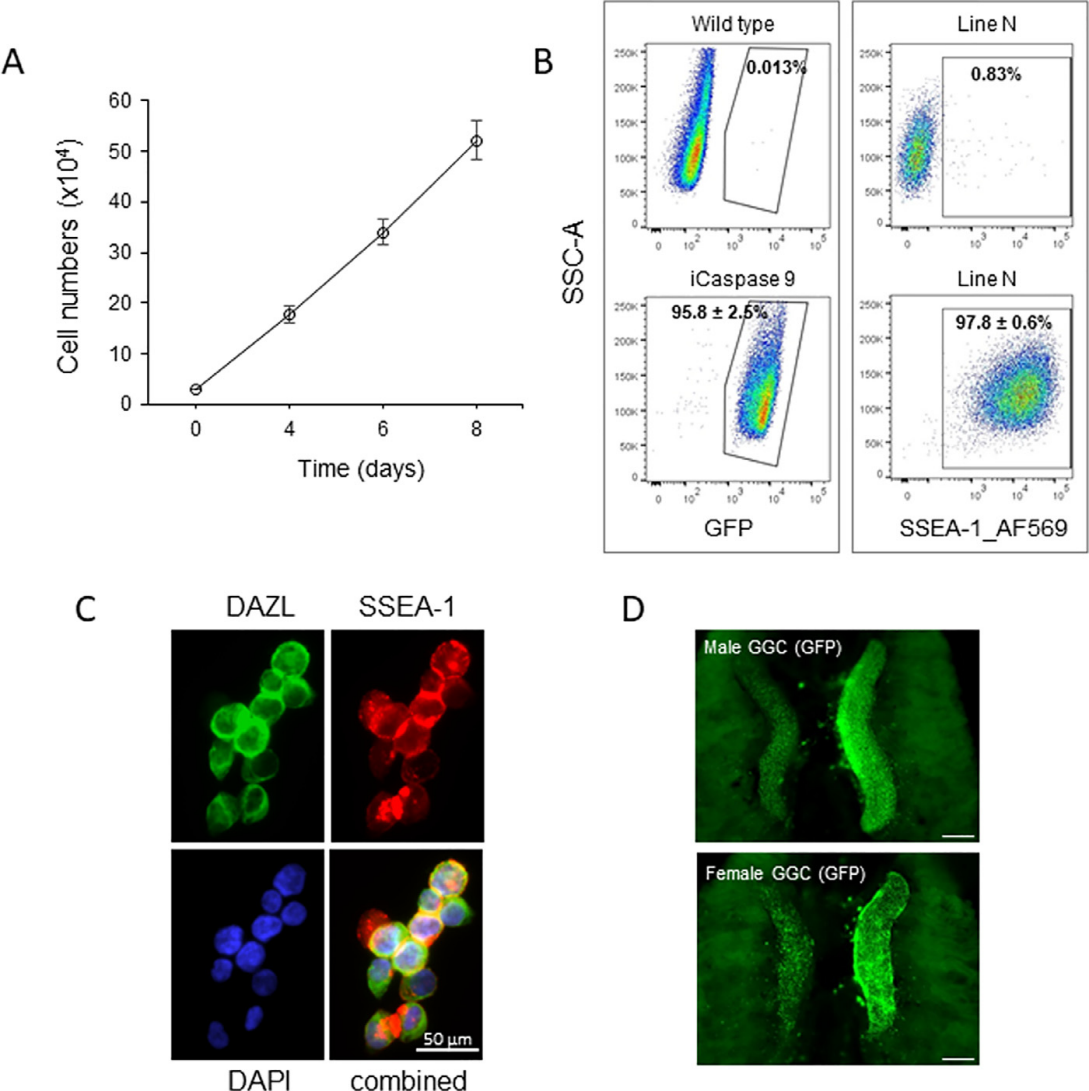
Characteristics of cultured GGCs derived from frozen gonads. (A) The growth parameters of gonadal germ cells, 8 female germ cell derivations were tested and cell number start with 3 × 10^4^ cells, the data represent average cell number at each time point, error bar is S.E.M. (B) Flow cytometric analysis of germ cell homogeneity, cell derivations are from wild type or iCaspase 9 transgenic embryos (n = 9, left panel), germ cell expressing GFP and cells from inbred line N embryos (n = 8, right panel), the cells stained by SSEA-1 or isotype control antibody, the GFP^+^ or SSEA-1^+^ cells were gated. (C) Expression of germ cell markers by cultured gonadal germ cells from Line N birds. (D) Re-colonization of host embryonic gonads by GFP^+^ gonadal germ cell derivations. Male or female GGCs were injected into 2.5 d wildtype host embryos. Chimeras were analyzed at d 8, n = 2. Scale bar = 1 mm.

We measured the growth rate of established GGC derivations from frozen gonads during the derivation process. Figure 2A shows a linear growth pattern when plotting total cell number against growth time. The cell number was increased 6-fold after the first 4 d of culture then slightly slowed down with 1.9 folds increase at d 4-6 and 1.5-folds increase at d 6-8, and a cell doubling time of 1.94 d in 8 d'culture. When the cells grew until the expected number (>10^5^) was achieved, the cell purity was analyzed by flow cytometry. The results shows that 95% cells among GGC derivations from iCaspase 9 embryos were GFP positive in comparison with wild type GGCs (non GFP expression). Immunostaining of GGCs from line N embryos resulted in 97% cells positive for SSEA-1 when compared with isotype control staining, indicating that the cultured cells are highly pure germ cells. The fluorescent imaging further demonstrates that the GGC derivations express the PGC markers SSEA1 and DAZL (Figure 2C). Next, GGCs from GFP transgenic embryos were injected into non-GFP recipients in order to visualize the competency of cultured GGC migration. As shown in Figure 2D, the donor cells migrated to and colonised the host gonads in chimeric embryos.

## DISCUSSION

When avian GGCs colonise the gonadal anlagen, their population expands rapidly and reaches over 10,000 cells at embryonic developmental stage HH35 (d 9–10) for chicken. Accumulated evidence shows that isolated gonadal germ cells can re-populate a host's gonads when re-introducing them via the dorsal aorta of a 2.5 day-old embryo (Tajima et al., 1998; Nakajima et al., 2022; Park et al., 2003; Hu et al., 2022), indicating that GGC from chicken embryos at mid developmental stages (< 10 d) retain primordial germ cell properties and migratory competency through the circulatory system. This knowledge supports the finding that GGCs can be a source for avian germ cell derivations, potential application in biobanking of avian species, and generation of genetically modified avian species. Reconstitution of chicks from the frozen material will depend on access to sterile surrogate host embryos. It is hoped that further sterile recipient chicken lines will be developed and improved chemical and physical methods for embryonic germ cell ablation of surrogate host embryos are also developed.

A recent study showed that GGC can be quickly discharged from whole gonadal tissues of d 7 embryos when incubated in warm PBS at 37°C (Nakajima et al., 2022). Using this knowledge, we incubated whole gonads from 9 to 10 day-old embryos in FAOT, a low calcium defined PGC growth medium in hope that GGCs shed from gonads will seed germ cells for long term culture. An advantage of culturing germ cells from whole gonadal tissues instead of dissociated gonadal cells will simplify the process and is suitable for culturing individual gonadal tissue from a large number of embryos in a less labor-intensive manner. Using iCaspase 9 transgenic embryos whose germ cells are labelled with a GFP transgene (Ballantyne et al., 2021), we could readily identify the GGCs discharged from whole tissues and visualize cell proliferation. After 20 h culture, a pair of female gonads (10 day-old embryo) yields 7,000 cells, 40% of total germ cells (18,000 cells), and a pair of male gonads yields 1,500 cells, 10% of total germ cells (14,000 cells) present at this stage (Hu et al., 2022; Yang et al., 2018). Although the heterogeneity of this result is not fully understood for gonadal germ cells from d 10 embryos, 1 possibility is that the discharged GGCs may possess PGC characteristics, whereas the GGCs remaining inside gonads may reflect a differentiated status. Consequently, the male gonads released less cells because male germ cells reside deeply inside gonadal tissues of d 10 embryos and, in contrast, female gonads release more germ cells, because the cells are located in the cortex, the outermost layer of gonads, and easily migrate from the whole tissues (Nakajima et al., 2011). As expected, the left gonad produced more germ cells than that of right gonads in both sexes, probably because the population of migratory competent germ cells is higher in left gonads (Naito et al., 2009), which is a consistent observation when culturing day 7 embryos (Nakajima et al., 2011). Nakajima et al. (2011) observed that incubating day 7 embryonic gonads in PBS (37°C), generated about 2500 GGC after 12 h incubation but after this point cell death greatly reduced the number of surviving GGC. This number is consistent with our finding with the culture of day10 embryonic gonads, suggesting the germ cells start to differentiate once they reside inside gonads, but not in a synchronous pattern. Similar to fresh tissues, the GGC can also migrate out when culturing frozen whole gonadal tissues in the same conditions. The left gonads from both sexes provided higher numbers of GGC than that of right gonads.

The freeze/thaw process had more subtle effects on the GGC numbers released from left gonadal tissues. Both fresh and frozen gonadal tissues produced comparative number of live GGCs in both sex, except for lower numbers of cells from frozen right gonads than that of cells from fresh right gonads. This information suggests that left female gonads will be more important than right gonads in biobanking of avian species.

Contaminating somatic cells, such as gonadal somatic cells and red blood cells, are also present in GGC cultures. During the culture of day 7 embryonic gonads, the GGC purity decreases from 51% at 1.5 h incubation to 20% at 24 h incubation (Nakajima et al., 2011). The 20 h culture of day 10 embryonic gonads produced a 10% to 70% GGC purity, with contaminating cells predominantly being red blood cells. However, during the following culture period, the red blood cells lyse and any contaminating somatic cells adhere to the culture plate. The GGCs remain in suspension and can be separated easily, eventually resulting in over 95% purity for the GGC derivations, uniformly expressing germ cell specific markers. Injection of these established GGCs into 2.5 day recipient embryo further demonstrates their germ cell capacity to migrate and colonise the host embryo's gonads.

Cryopreservation is a harsh process, with dramatic changes in cellular osmolality during the freeze/thaw process and often results in structural and physiologic damage or death. Freezing whole gonadal tissues from 10-d embryos seemingly protects GGCs better than freezing dissociated gonadal cells (Hu et al., 2022). The GGC derivation rates from the frozen gonadal tissues are high, but variable among the chicken lines. Nakajima et al. (2022) showed GGC that migrate from the gonad into low calcium medium can be directly cryopreserved and used for injections into surrogate host embryos. Future research is needed to directly compare the in vitro culturing of GGC cryopreserved in these 2 manners.

When the effect of the chicken line is considered, it is interesting to note that for the GFP line, the male gonads had a lower derivation rate (5 out of 12 individual gonads from 6 embryos successfully cultured), which was probably due to failure in culture of the right gonads as mentioned previously. In contrast, the Hyline genetic background may be the reason for a robust derivation rate (100%) from iCaspase 9 male gonads which were frozen for almost 2.5 years. Female gonads had higher derivation rates than that of male gonads in inbred line N and cross-bred Light Sussex, probably due to left gonads shedding more GGCs at the beginning of culture. However, for gonads from line J, although the tissues were stored in liquid nitrogen, GGC derivation rate is very low, which is consistent with the rate of PGC derivation from HH16 embryonic bloods, 42% (14 out of 33 embryos) success for male, 0% (0 out of 27 embryos) for female (Sunil Nandi, unpublished data), suggesting chicken line is also an important factor affecting the germ cells in *in vitro* culture. Additionally, the freeze/thaw processes do increase the *in vitro* culture time needed to reach expected cell yield when compared with fresh tissues. This is logical because the GGCs may require time for equilibration to the cell culture environment and time for cellular repair (e.g. cytoskeleton reorganization, membrane repair) before they are able to resume the cell cycle. This suggests that use of a different cryopreservation media may be warranted, which should allow indefinite cryopreservation of the frozen gonadal material. In our experiments, we used a commercial ES cell freezing medium but did not assay standard 5 or 10% DMSO/ FBS-containing freezing media. Also, dissection of embryonic gonads into 2 to 3 pieces may aid the freezing process and the post-thawing migration of gonadal cells into the culture milieu.

To conclude, this simplified method for culturing of GGC from cryopreserved embryonic gonads may be a supplementary approach for safeguarding the genetic resources and genetic diversity of avian species. Cultured GGC, derived from cryopreserved gonads, can be used for breed regeneration and subsequently cryopreserved. The application of this method could reduce the number of live birds in either research facilities or conservation centers for maintenance of chicken breeds. On-line accessible protocols and videos, stakeholder workshops, and training sessions will be needed to transfer this cryopreservation technology to low- and middle-income countries (Gitari, 2024; Cirdes Bobo-Dioulasso, 2024). Culturing of PGC or GGC from nonchicken bird species is currently not possible, yet efforts in this area have increased (Chen et al., 2019). We propose that biobanking of embryonic gonads from avian species, both at risk and common, would be prudent in foresight of future technological developments.

## DISCLOSURES

All authors declare no conflict of interest.
